# Comparative Analysis of Static and Viscoelastic Mechanical Behavior of Different Luting Material Categories after Aging

**DOI:** 10.3390/ma14061452

**Published:** 2021-03-16

**Authors:** Nicoleta Ilie

**Affiliations:** Department of Conservative Dentistry and Periodontology, University Hospital, Ludwig Maximilians University, Munich Goethestr. 70, D-80336 Munich, Germany; nilie@dent.med.uni-muenchen.de; Tel.: +49-89-44005-9412; Fax: +49-89-44005-9302

**Keywords:** luting materials, flexural strength, modulus, interfacial fracture toughness, dynamic mechanical analysis, viscoelasticity

## Abstract

The longevity of indirect restorations is primarily determined by the appropriate selection of the luting material. The function of a luting material is to seal the restoration and hold it in place for the time required for service. The mechanical behavior of luting materials and in particular their aging behavior, therefore, play a decisive role. The study provides a comparative analysis of the static and dynamic mechanical behavior of the most commonly used luting material categories—zinc phosphate cement, glass–ionomer cement, resin-modified glass–ionomer cement, resin-based composites, and self-adhesive resin-based composites—and their aging behavior. It also takes into account that luting materials are viscoelastic materials, i.e., materials that respond to external loading in a way that lies between an elastic solid and a viscous liquid. Flexural strength and modulus were determined in a three-point bending test followed by fractography analysis. The quasi-static and viscoelastic behavior was analyzed by a depth-sensing indentation test provided with a dynamic mechanical analysis (DMA) module at 20 different frequencies (1–50 Hz). The fracture toughness was evaluated in a notchless triangular prism (NTP) test. Material type exhibits the strongest influence on all measured properties, while the effect of aging becomes more evident in the material reliability. The variation of the viscoelastic parameters with aging reflects cement maturation or polymer plasticization.

## 1. Introduction

Luting materials are an essential type of restorative materials that must fulfill a variety of functions in a restoration. They are used to permanently or temporarily fix restorations to prepared tooth structures or implant abutments, while sealing the joint between dentin/enamel/abutment and the restoration, securing the position of the restoration under occlusal loads, and transferring the chewing force from the restoration to the tooth/abutment [1]. In addition to the functions of establishing a connection between restoration and tooth/abutment and not harm the tooth or tissue [2], luting materials have to withstand in the oral cavity high mechanical loads, fatigue, and chemical and enzymatic attacks. These complex tasks require high standards in the selection of a suitable luting material [1] and have led to the use of material categories that differ in their chemical composition [2,3], setting reaction, and ultimately, physical and mechanical properties [1,4].

Historically, zinc oxide phosphate cements are among the oldest luting materials. The phosphate cement is considered to be the cheapest type of cementation and can look back on more than 100 years of clinical use. It is regarded as a biocompatible material, while no allergic, toxic, or mutagenic effects are known in clinical use. However, phosphoric acid is viewed critically, as it can damage the pulp if its content in the cement is high or the remaining dentin is too thin [1]. Adjustments of the setting reaction and the use of quick-setting cements helped to alleviate this disadvantage [5].

Glass–ionomer cements (GICs) and their derivative, the resin-modified glass ionomer cements (RMGICs), are popular and proven permanent luting materials [6]. GICs, the product of the reaction between ion-leachable glasses and an aqueous solution of (poly)acrylic acid and co-polymers, were developed by Wilson and Kent more than 50 years ago [7]. They are one of the few restorative materials that can chemically bond to the tooth structure without an intermediary agent. This is achieved through chelation with the calcium ions in dentin and tooth enamel. In addition, they offer good translucency, increased cariostatic potential due to the fluoride release, and good clinical performance in cementing metal and ceramic restorations also after 10–20 years of clinical function [6]. The GIC is less soluble than the phosphate cement but is only moderately acid-resistant [7]. In clinical use, however, there is no noticeable advantage of GIC over the zinc oxide phosphate cements [6]. The addition of hydrophilic monomers, usually 2-hydroxyethyl methacrylate (HEMA), to conventional GIC led to a new category of materials, the RMGICs, which was developed to overcome the high solubility of GICs and to improve their poor mechanical properties and stability during the initial setting reaction [6]. Curing is achieved in RMGICs by two different mechanisms. A slow acid-base reaction is juxtaposed to a fast radical polymerization; the latter typically being promoted by the action of visible light. RMGIC luting materials have been shown to be clinically comparable to zinc phosphate cements [8].

Resin-based composite (RBC) luting materials were introduced in the 1970s and are widely used [9]. In conjunction with an adhesive, they enable good mechanical stability of the restoration and, due to the enhanced translucency, offer improved aesthetic properties when compared to GIC or zinc oxide phosphate cements [10]. RBC luting materials are used for restorations with non-retentive preparation, such as adhesive luted bridges or veneers, and for brittle materials, such as glass ceramics. The setting reaction in this type of material can be initiated either chemically, with visible light, or by a combination of both (dual-curing). Another step to speed up the restoration process is to forego the use of an adhesive and modify the luting RBC to enable self-adhesive properties. There is little or no clinical data for most self-adhesive RBC luting materials, although they have been on the market for several years [3].

This great variation of luting materials justifies the question of what essential differences in their mechanical behavior can be observed and how these can be transferred to a clinical situation. Aging would also be an important means of simulating clinical conditions. In addition, all of the materials described above show a viscoelastic behavior that usually remains unexamined since the essential investigation methods are based on quasi-static tests. The aim of this study was therefore to carry out a comparative investigation of a representative number of luting material categories under static and dynamic conditions, including artificial aging.

The tested null hypotheses were that examined luting materials (zinc phosphate cements, glass ionomer cements, resin-modified glass ionomer cements, resin-based composites, and self-adhesive resin-based composites) will behave similarly with regard to (a) strength, elastic modulus, and fracture pattern; (b) viscoelastic (storage modulus E′, loss modulus E″, and loss tangent tan δ) and quasi-static mechanical properties (indentation hardness H_IT_, indentation modulus, E_IT_), in addition to their variation pattern with the frequency (1 to 50 Hz); (c) fracture toughness; and d) aging.

## 2. Materials and Methods

Five materials belonging to the most often used luting material categories—dual-cured resin-based composites (RBCs); dual-cured, self-adhesive dual-cured RBC; glass ionomer cement (GIC); resin-modified glass ionomer cement (RMGIC); and temporary zinc phosphate cement—have been analyzed (Table 1). Their properties were assessed at 24 h post setting/curing and after thermal aging (TC, 10,000 thermocycles between 5 °C and 55 °C at a dwell time of 30 s per temperature and a transfer time of 10 s, Willytec, Dental Research Division, Munich, Germany). Flexural strength (FS) and flexural modulus (FM) were determined in a three-point bending test followed by a fractography analysis. The quasi-static and viscoelastic behavior of the materials was determined by a depth-sensing indentation test equipped with a dynamic mechanical analysis (DMA) module at frequencies from 1 Hz to50 Hz. The fracture toughness was assessed in a notchless triangular prism (NTP) test. Specimens made out of the dual-cured RBCs and the RMGIC were light-cured with a violet-blue light emitted diode (LED) light-curing unit (Bluephase^®^ Style; Ivoclar Vivadent; Schaan, Liechtenstein; Irradiance = 1386 mW/cm, as assessed by a spectrophotometer, MARC System, Bluelight analytics, Halifax, NS, Canada).

### 2.1. Three-Point Bending Test 

The flexural strength (FS) and flexural modulus (FM) were measured in a three-point bending test, in accordance with ISO 4049:2009 [11]. Therefore, 200 (*n* = 20) specimens were prepared by compressing the material between two glass plates with intermediate polyacetate sheets, separated by a Teflon mold having an internal dimension of 2 mm × 2 mm × 18 mm. Specimens made out of the dual-cured RBCs and the RMGIC were light-cured for 20 s in accordance with ISO 4049:2009 [11]. Cement specimens were stored in 100% humidity for 1 h in their mold and then removed from the mold and stored for an additional 23 h in distilled water 37 °C to fulfill a 24 h storage interval. RBC specimens were stored after preparation in distilled water at 37 °C for 24 h. Half of the specimens were tested after 24 h of storage, and the other half were subjected to additional thermal aging before measurement. The samples were loaded to failure in a universal testing machine (Z 2.5, Zwick/Roell, Ulm, Germany) at a crosshead speed of 0.5 mm/min. The three-point bending test device was constructed according to the guidelines of NIST (National Institute of Standards and Technology) No. 4877 with a span of 12 mm. The specimens were immersed in distilled water at room temperature during the test. 

### 2.2. Fractographic Analysis

The fractured surfaces were examined in a stereomicroscope (Stemi 508, Carl Zeiss AG, Oberkochen, Germany) in order to determine the fracture pattern. The surfaces were photographed using a microscope extension camera (Axiocam 305 color, Carl Zeiss AG, Oberkochen, Germany). The origin of the fracture was determined and divided into one of four different groups (sub-surface-, edge-, corner- or plain-failure). 

### 2.3. Instrumented Indentation Test (IIT)

To determine the quasi-static and viscoelastic behavior of the analyzed materials a depth-sensing indentation test extended with a DMA module (FISCHERSCOPE^®^ HM2000, Helmut Fischer, Sindelfingen, Germany) was used. The method is described in more detail elsewhere [12]. The fragments resulting from the bending test were used for this measurement (*n* = 6). Specimens were therefore wet-ground by means of an automatic grinding machine (EXAKT 400CS Micro Grinding System EXAKT Technologies Inc. Oklahoma City, OK, USA) while using silicon carbide sandpaper (grit size p1200, p2500, and p4000, LECO Corporation, St. Joseph, MI, USA). The surface preparation was finished with a polishing procedure for 2–3 min with a diamond suspension (mean grain size: 1 µm).

#### 2.3.1. Quasi-Static Indentation Test

A requirement for the DMA analysis is the parameter µ_IT_ (=W_elast_/W_total_), which was determined by ISO 14577 [13] by means of an automated nano-indenter (FISCHERSCOPE^®^ HM2000) using a Vickers diamond tip. For this purpose, one measurement was performed at random per sample (*n* = 6). The test uses an indentation force that increased and decreased with a constant velocity from 0.4 mN to 1000 mN within 20 s while recording the corresponding indentation depth. The measurement enables the calculation of both elastic and plastic deformation works. The sum of the two is the total mechanical work, defined as W = ∫ Fdh (F = load; h = indentation depth). During indentation, only part of this work is consumed as plastic deformation (W_plast_), while the rest represents the elastic reverse deformation W_elastic_ that is released.

#### 2.3.2. Dynamic Mechanical Analysis (DMA)

The dynamic mechanical analysis uses a sinusoidal oscillation of small magnitude, which is superposed on a quasi-static force (F_max_ = 1000 mN). The amplitude of this oscillation (five nm) takes into account sample deformation in the linear viscoelastic range. The samples (*n* = 6) were exposed to 20 different frequencies in the range 1–50 Hz (1.0 Hz; 1.2 Hz; 1.5 Hz; 1.9 Hz; 2.3 Hz; 2.8 Hz; 3.4 Hz; 4.2 Hz; 5.2 Hz; 6.4 Hz; 7.8 Hz; 9.6 Hz; 11.8 Hz; 14.5 Hz; 17.9 Hz; 21.9 Hz; 26.9 Hz; 31.1 Hz; 40.7 Hz; 50.0 Hz). The selected frequencies are denser in the lower range in order to do justice to the chewing activity in humans, which mainly occurs in the range of 0.94 Hz (5th (th) percentile) and 2.17 Hz (95 (th) percentile) [14]. Frequencies then increase logarithmically up to 50 Hz in order to enable identifying structural differences in the analyzed materials. Overall, 10 measurements were performed for each frequency and indentation (six per specimen). The sinusoidal oscillation generates oscillations on the displacement signal with a phase angle δ, which enables calculating the storage (E′) and the loss moduli (E″). E′ represents the elastic response of material behavior, whereas E″ describes the viscous material behavior. The tangents of the phase angle, δ, is defined as the loss factor (tan δ = E″/E′) and is a measure of the material damping behavior.

In addition to the viscoelastic parameters described above, the test enables further calculation of quasi-static parameters, such as the indentation hardness (H_IT_ (=F_max_/A_p_)) and the indentation modulus (E_IT_) [13]. 

### 2.4. Notchless Triangular Prism (NTP) Test

The fracture toughness procedure uses a 6 mm × 6 mm × 6 mm × 12 mm notchless triangular prism (NTP) test specimen (*n* = 12), accordingly to the test developed and described by Ruse et al. [15]. To manufacture the triangular prism specimens, the luting materials were compressed between two glass plates separated by a split Teflon mold. The light curing in the materials that required this additional procedure was carried out for 20 s. Test samples were additionally polymerized along their prismatic surfaces and then stored in distilled water at 37 °C for 24 h. Cement specimens were removed from the mold one hour after setting under compression at 37 °C in a vapor-saturated atmosphere and then stored in distilled water at 37 °C for 23 h. After storage all specimens were subjected to thermal aging, as described above. For K_IC_ measurements, the specimen was fixed in one half of the specimen holder, a small defect was produced with a surgical blade (Ted Pella, Redding, CA, USA., USA), and the specimen was then fixed in the second half of the specimen holder, using the mounting block [16]. Specimens were then loaded in the NTP-test assembly in tensile at a crosshead speed of 0.1 mm/min in a universal testing machine (Zwick/Roell). The load and the displacement were constantly monitored and recorded. The maximum load at crack arrest or fracture (F_max_, in N) was used to calculate K_IC_, in MPa·m^1/2^, using the equation below:

(1) $$K_{IC}=Y_{min}^{*}\frac{F_{max}}{D\sqrt{W}}$$

where Y_min_* = 28, *D* = 12 mm and *W* = 10.4 mm [15]. 

### 2.5. Statistical Analyses

The distribution of the variables was tested with the Shapiro–Wilk procedure. Equality of variance was determined by way of Levene’s test. Since the variables were normally distributed, a parametric approach was used. The parameters of interest (flexural strength *FS*, flexural modulus *FM*, storage modulus *E*′, loss modulus *E*″, loss tangent tan *δ,* indentation hardness *H_IT_,* indentation modulus *E_IT_*, fracture toughness *K_IC_*) were compared by means of a multifactor analysis of variance. In addition, one- and multiple-way analysis of variance (ANOVA) and Tukey honestly significant difference (HSD) post hoc test (α = 0.05) with an alpha risk set at 5% was used. A multivariate analysis (general linear model) quantified the effect of parameters *material type* and *aging* (additional *frequency* for the parameters determined in the instrumented indentation test) and their interaction products on the analyzed properties. The partial eta-squared indicate the ratio of variance associated with an effect. Larger values of partial eta-squared (η_P_^2^) indicate a greater amount of variation accounted for by the model (SPSS Inc. Version 25.0, Chicago, IL, USA). 

For FS data, a Weibull analysis was performed. The Weibull model represents a common empirical expression for the cumulative probability of failure P at applied stress σ is [17] as follows:

(2) $$P_{f}(\sigma_{c})=1-\exp\left[-{\left(\frac{\sigma_{c}}{\sigma_{0}}\right)}^{m}\right]$$

In this equation, $\sigma_{c}$ represents the measured strength, m the Weibull modulus, and $\sigma_{0}$ the characteristic strength. Latter is defined as the stress at which the probability of failure is 0.63. The double logarithm of this expression is $\ln\ln\frac{1}{1-P}=m\ln\sigma_{c}-m\ln\sigma_{0}$. By plotting ln ln(1/(1 − P)) versus ln $\sigma_{c}$, a straight line, with the upward gradient m results, while the point of intersection with the x-axes gives the logarithm of the characteristic strength [17].

## 3. Results

### 3.1. Three-Point Bending Test 

**Figure 1:**
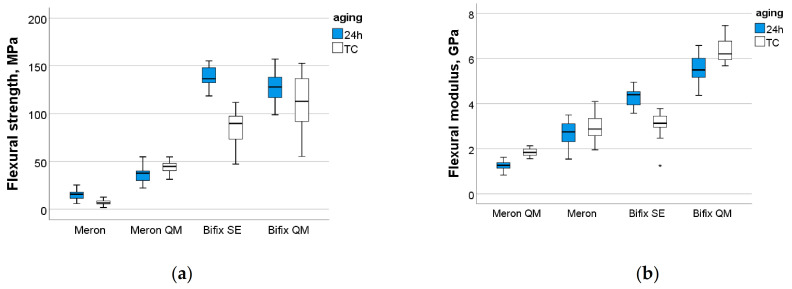
Flexural strength (**a**) and modulus (**b**) as a function of luting material and aging conditions.

**Figure 2:**
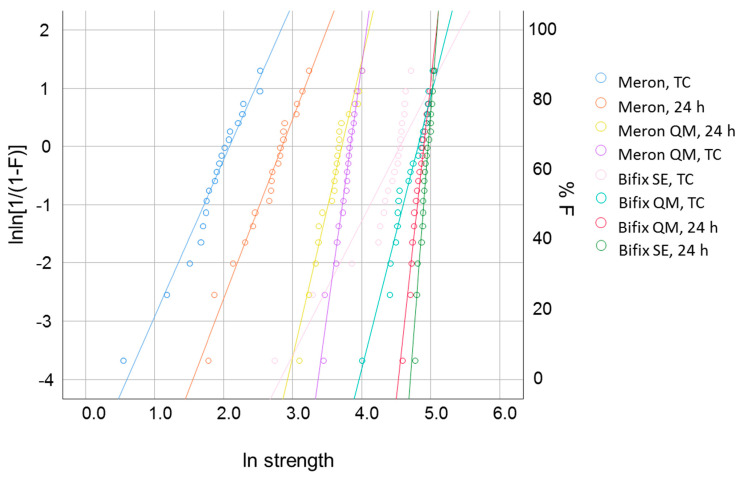
Weibull distribution as a function of luting material and aging conditions.

Parameters measured in the three-point bending test and the corresponded Weibull moduli are illustrated in Table 2 and Figure 1 and Figure 2. Samples made from Provicol failed when inserted into the testing device, while those exposed to thermal aging failed during this procedure.

A multifactorial analysis showed a significant and strong influence of the parameter *material* both on FS (*p* < 0.001, η_P_^2^ = 0.903) and on FM (*p* < 0.001, η_P_^2^ = 0.878). In contrast, the *aging* effect on FS was small (*p* < 0.001, η_P_^2^ = 0.274) and not significant on FM (*p* = 0.340). Their interaction product *material × aging* had a significant influence on both measured parameters.

After storage for 24 h, the FM and FS values decreased significantly in the material sequence Bifix SE < Bifix QM < Meron Plus QM < Meron. This sequence was maintained after aging, with the amendment that the FM of Bifix SE and Meron were statistically similar (*p* = 0.799).

Material’s reliability (m, Weibull modulus) was 24 h after setting highest in Bifix SE and decreased in the same sequence as mentioned above for FS. The reliability was strongly influenced by *aging* and was highest for Meron QM, followed by Bifix QM and the statistically similar group of Bifix SE and Meron QM. Both RBCs (Bifix QM and Bifix SE) lost tremendous reliability with aging, while the loss for Bifix SE was higher. Meron retained its initial poor reliability after aging, while Meron Plus QM was the only material that showed a slight but significant increase in reliability after aging. The significance level shown above relates to the 95% confidence interval calculated for *m* (the 95% confidence interval results from the values 1.96 × Std. Error on both sides of the mean). 

### 3.2. Fractographic Analysis

**Figure 3:**
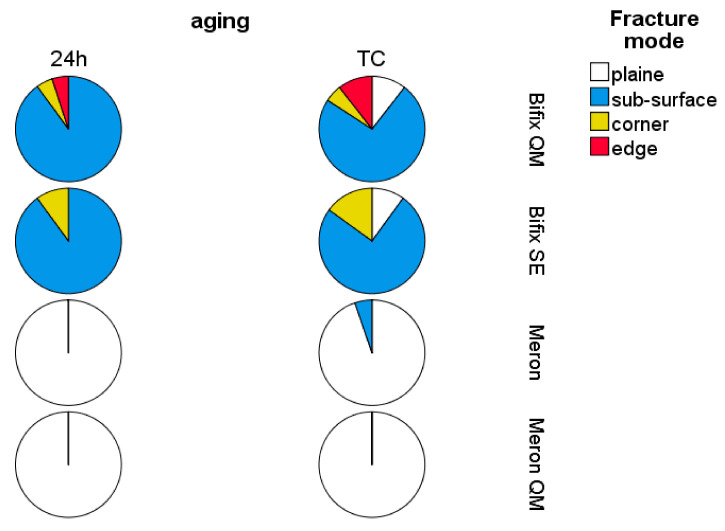
Fracture modes as a function of luting material and aging conditions.

A fairly even distribution between the less brittle fracture mode entitled plain (52.2%) and sub-surface fracture (41.5%) was observed, with corner (4.4%) and edge fractures (1.9%) being the least likely modes of fracture (Figure 3). However, there are two different types of behavior evident, which are material type characteristic. Sub-surface (80%) was the most common mode of fracture in the RBC materials (Bifix QM, Bifix SE), while almost all GIC specimens (Meron and Meron QM) showed a plain fracture (only one Meron sample failed due to a sub-surface defect).

A one way ANOVA, followed by a Tukey’s post-hoc test, displayed a significant decrease in FS when fracture occurred in the plain fracture mode (FS = 32 MPa) compared to the other described fracture mechanisms (*p* < 0.05), which formed a homogeneous subset (*p* = 0.625, FS = 99 MPa to 115 MPa). The differentiation of the fracture modes in relation to FM was clearer. FM decreases in the following sequence of fracture modes (sub-surface, edge fractures, *p* = 0.062) < (sub-surface, corner fractures, *p* = 0.436) < (corner, plain fractures, *p* = 0.07).

### 3.3. Instrumented Indentation Test (IIT)

The quasi-static test made it possible to determine the ratio of the reverse elastic deformation work of indentation (W_elast_) and the total mechanical work of indentation, a parameter that is mandatory for the dynamic mechanical analysis. A multifactorial analysis showed a significant and strong influence of the parameter *material* on μ_IT_ (*p* < 0.001, η_P_^2^ = 0.940), while the effect of *aging* was mild (*p* = 0.02, η_P_^2^ = 0.157). Accordingly, μ_IT_ increased from 27% to 51% in the sequence Meron (24 h and TC, *p* = 0.729) < Meron QM (24 h) < (Meron QM (24 h); Bifix SE (24 h and TC), *p* = 0.99) < (Bifix QM (24 h and TC), *p* = 0.08). In contrast, the total indentation work (W_total_) was strongly influenced by *aging* (*p* < 0.001, η_P_^2^ = 0.767), was lowest in BifixQM (2.9 μJ), and increased up to 4.5 μJ in Meron. 

**Figure 4:**
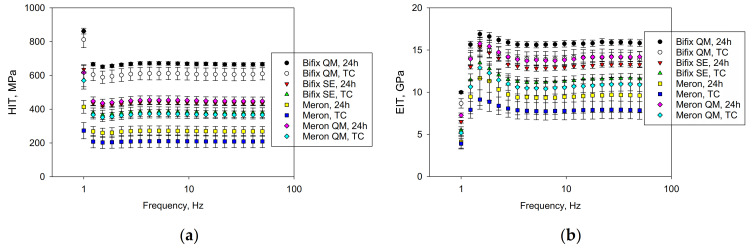
Variation of (**a**) indentation hardness H_IT_ and (**b**) indentation modulus E_IT_ over the frequency range 1–50 Hz for the materials analyzed.

The quasi-static parameter H_IT_ increased significantly in the sequence Meron, TC < Meron, 24 h < Meron QM, TC < Bifix SE, TC < Bifix SE, 24 h < Meron QM, 24 h < Bifix QM, TC < Bifix QM, 24 h (Figure 4a), while values measured in the RBC Bifix QM were up to three times higher than in the GIC (Meron). The highest H_IT_ values were recorded for all materials at a frequency of 1 Hz. With the following frequency, the H_IT_ values dropped to a plateau. This jump was smaller in the GIC than in the resinous materials. Aging significantly reduced the H_IT_ in all materials and at all frequencies.

The difference between the parameters E_IT_ and E′ is small. The material sequence regarding E_IT_ and E′ is similar, as described above for H_IT_. In this sequence, only Meron QM, 24 h and Bifix QM TC are statistically similar (*p* = 0.99). In contrast to the variation pattern with frequency identified for H_IT_, the lowest values were observed with the lowest frequency used in this test (1 Hz). The values increase to a maximum within the next two frequency steps and then drop to a plateau up to a frequency of 3.4 Hz (Figure 4, right).

**Figure 5:**
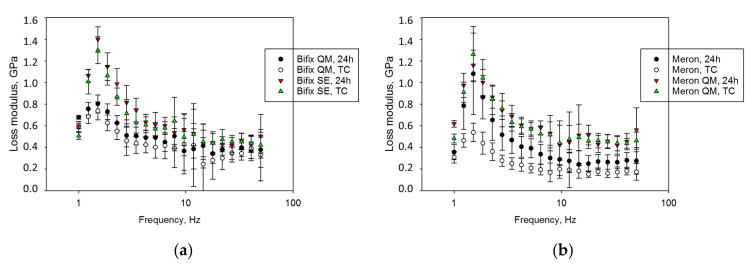
Variation of the loss modulus (E″) over the frequency range 1–50 Hz for (**a**) Bifix QM and Bifix SE; (**b**) Meron and Meron QM.

The loss modulus (E″) increases with the frequency in the firsts three analyzed frequencies to a maximum and shows a frequency-independent plateau only at frequencies above 10 Hz (Figure 5). E″ was lowest in the aged GIC, while it was highest in Bifix SE and Meron QM. With the exception of the GIC, aging did not affect E″ at any frequency analyzed.

**Figure 6:**
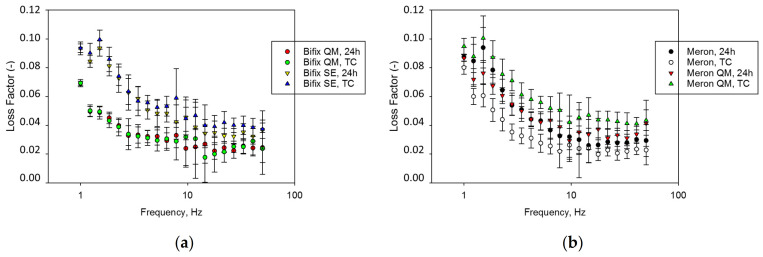
Variation of the loss factor (tan δ) over the frequency range 1–50 Hz for (**a**) Bifix QM and Bifix SE; (**b**) Meron and Meron QM.

The loss factor (tan δ) decreased steadily up to 11.8 Hz to a plateau in all analyzed materials (Figure 6). Over the analyzed frequencies, tan δ decreased in the sequence (Bifix QM, TC; Bifix QM, 24 h; Meron, TC; *p* = 0.99) < (Meron, 24 h; Meron QM, 24 h; *p* = 0.99) < Bifix SE, 24 h < (Bifix SE, TC; Meron QM, TC; *p* = 0.961).

The parameters *material*, *aging,* and *frequency,* and their binary and ternary interaction products exert a significant effect on the measured properties. The parameter *material* influenced H_IT_ the most (*p* < 0.001, η_P_^2^ = 0.965), followed by E_IT_ and E′ (η_P_^2^ = 0.522 for both), E″ (η_P_^2^ = 0.522), and tan δ (η_P_^2^ = 0.515). The *frequency* exerted a comparable influence on all parameters in the sequence tan δ (η_P_^2^ = 0.784), E″ (η_P_^2^ = 0.718), E′ (η_P_^2^ = 0.662), E_IT_ (η_P_^2^ = 0.661), and H_IT_ (η_P_^2^ = 0.656). The effect of *aging* was very low on E″ (η_P_^2^ = 0.08), and tan δ (η_P_^2^ = 0.003), while moderate on the other parameters (E′: η_P_^2^ = 0.580; E′: η_P_^2^ = 0.530; E_IT_: η_P_^2^ = 0.529).

### 3.4. Notchless Triangular Prism (NTP) Test

The fracture toughness decreased significantly in the sequence Bifix SE > Bifix QM > Meron QM plus > Meron (Table 3), while samples made from Provicol did not survive thermal aging. Two different stages of pre-failure were identified in the samples analyzed: samples that failed during the thermal aging process and samples that failed during fixation in the test device. Pre-failure due to thermal aging was only observed with Provicol samples, all of which failed. Since the samples were not analyzed until the end of the 10,000 thermal cycles, the exact time of failure cannot be given. Two samples failed when mounted in the test device (Bifix QM). They were not included in the mean calculation and statistical analysis.

## 4. Discussion

The selection of a suitable luting material depends on the specific requirements of a clinical situation. Important selection criteria encompass the material used for the indirect restoration, the ability of the luting material to create a stable connection between the restoration and the tooth/implant abutment, the mechanical behavior of the luting materials, and even their color and translucency [1]. The present study has focused on one, albeit a very complex aspect within these selection criteria, namely, the mechanical behavior. It offers a comparative study of luting materials belonging to five main material categories and their aging behavior. The study also takes into account that luting materials are viscoelastic materials, i.e., materials that react to external forces in a way that lies between the behavior of an elastic solid and a viscous liquid and thus must be analyzed accordingly [18]. The luting materials analyzed—zinc phosphate cements, glass ionomer cements, resin-modified glass ionomer cements, resin-based composites, and self-adhesive resin-based composites—behaved differently when exposed to static or oscillatory loads, or when they were aged. Therefore, all null hypotheses must be rejected.

The analyzed zinc phosphate cement, Provicol, is a temporary luting material. These materials must ensure that the luted restoration is secured and can be easily removed. From a chemical point of view, there is less potential for interaction with the luting material for both the tooth and the restoration, while the micromechanical retention is weak. Analyzed in bulk, the samples were still intact after setting and demolding but too weak to withstand placement in the three-point bending test, the instrumented indentation test, the NTP test, or during thermal aging. Since there is no mechanical stress during thermal aging, the disintegration of the specimen during this procedure is attributed to high water sorption or higher solubility due to the calcium hydroxide, which is an additive for the analyzed brand. Calcium hydroxide is frequently used in these cements because of its bacteriostatic effect and the ability to mediate the release of growth factors and other bioactive molecules from the dentine and thus induce dentine regeneration [19]. 

The glass ionomer luting cement Meron was the material with the lowest mechanical properties. It is a brittle material, as was confirmed by the measured low fracture toughness. FS and FM were also low. The glass ionomer (polyalkenoate) cement sets by the reaction of basic, powdery glasses (calcium–aluminum–fluoro–silicate glasses) with polyacrylic acid. This acid-base reaction is sensitive to both moisture (leaching of ions) and dehydration (loss of water), resulting in special treatment of the samples in the first hour after preparation, as described in the Materials and Methods section. One would have expected these properties to increase with aging as a result of cement maturation, but this was not the case. This behavior must be related to the inherent flaws in the material, particularly pores, as observed in abundance in the examined fractured specimens that may have initiated quick fracture. Fractography analysis performed in specimens, which was broken in bending at a low-stress level, showed a fairly flat but rough fracture surface due to the coarse microstructure and irregularities in the microstructure. In samples that were analyzed 24 h after setting, the fracture mirror could not be identified, while the origin of fracture can take on the character of porous areas because edges and corners remained intact. The fractured surface changed to a pronounced brittle appearance after thermal aging. The coarse microstructure and the irregularities imped also in this case an exact differentiation of the origin of fracture. However, Wallner lines became sporadically visible, marking a temporary deviation of the crack front out of the plane caused by the passage through a region with a locally shifted stress field due to the aforementioned porosities [20]. The data measured in the three-point bending test are validated by the quasi-static parameter H_IT_ and E_IT_, which follow a similar pattern of variation with the frequency as the other analyzed materials, but registered the lowest value over the entire frequency range. The data also confirm slightly lower values after thermal aging. In contrast to the above presented data, the viscoelastic parameters loss modulus and loss factor (tan δ) are significantly lower after aging than 24 h after setting, which is indicative of the maturation of the cement during aging. This behavior is specific to the analyzed GIC, while in all other analyzed materials tan δ remained insensitive or increased only slightly with aging, as will be discussed later. The loss factor tan δ is proportional to the ratio of energy lost to energy stored in one indentation cycle and approaches zero for ideal elastic materials. It can also be attributed to the material’s mechanical damping and its ability to absorb shock, i.e., its ability to reduce oscillation amplitudes through energy dissipation. The mechanism for energy dissipation in a material is associated with the internal friction caused either by grain or interphase boundary relaxation as proposed by Kê [21]. The viscoelastic behavior observed in the GIC must therefore be explained by the specific microstructure, the amount of the glass/cement phase, and the intrinsic flaws. In view of the fact that the material inherent defects in aged and not aged specimens must be comparable, differences in tan δ can be attributed to a change in the proportion of the glass/cement phase and thus to cement maturation.

The resin-modified glass–ionomer cement Meron Plus QM, is a dual-cured material that, in addition to the slower acid-base reaction, also sets by polymerization of the resin-phase. The radical polymerization was initiated here both chemically, by mixing the chemical catalyst when extruding the material, and by additional light curing. The rapid, initial hardening through photopolymerization of the methacrylate groups protects the cement against premature access to water and against dehydration. This confers the material quick stability and significantly superior mechanical properties compared to the GIC discussed above. The acid-base reaction occurs more slowly, as already described for GIC. The data confirm that Meron Plus QM is less brittle than the GIC. In addition, it was the only material that shows improved properties after aging. This may be interpreted as continuous maturation of the cement due to the ongoing acid-base reaction, while the polymer assured mechanical stability. On a microscale, maturation is undermined by plasticization of the polymer phase, as H_IT_ and E_IT_ are decreasing slightly with aging. While the loss modulus remained unchanged, the loss factor (tan δ) also increased slightly with aging, which confirms an increase in viscous behavior. Both the loss modulus and the loss factor are higher compared to the GIC, which can be attributed to the polymer content. Similar to the GIC, fractography analysis shows a fairly flat fracture surface, and no detectable fracture mirror or other brittle topographies. This behavior is related to the low-stress level at which the specimens were broken. The fracture surface is less rough compared to the GIC, but large cracks were visible in ¼ of the specimens, with a predilection in the compression zone. 

Bifix QM and Bifix SE are both methacrylate-based composites. As indicated in the safety data sheet, Bifix QM contains (2-hydroxyethyl methacrylate (HEMA) and bisphenol A-glycidyl methacrylate (Bis-GMA), while Bifix SE contains Bis-GMA, glycerol dimethacrylate, an acidic monomer, and hydroxypropyl methacrylate. Information on the amount and chemical composition of the filler is not available. With adequate light exposure of dual-cured RBC luting materials [22], as provided in the present study, the mechanical properties in RBCs are directly related to the amount of inorganic filler [23]. Bifix SE showed a higher FS but a lower FM compared to Bifix QM 24 h after setting, which is a clear indicator of lower filler amount and higher flexibility in the first. These properties dropped consistently after aging in Bifix SE, as a consequence of the more hydrophilic organic matrix and the higher water sorption. H_IT_ and E_IT_ values dropped in both materials during aging but remained higher in Bifix QM. The viscoelastic properties confirm the above line of reasoning and were higher in Bifix SE, as a consequence of the higher organic matrix amount. In addition, the damping ability steadily decreased to reach a plateau up to 11.8 Hz in all analyzed materials. From these observations, it can be concluded that the damping capacity of luting materials can be expanded over a broader frequency range, which is a good indication since restorations are subjected to cyclic masticatory loads that are transferred to the luting material.

## 5. Conclusions

The luting materials analyzed differed significantly in their mechanical properties. All showed viscoelastic behavior, while this behavior was material-specific and followed a different pattern of variation with aging. The temporary luting material disintegrated during thermal aging. The material type has the strongest influence on all measured properties, while the aging effect became evident in decreased reliability, thus highlighting the need to use Weibull analysis for strength data. The frequency exerted a comparable influence on all measured parameters, while the greatest variation occurred in the lower frequency range (1–3 Hz), which is the clinically relevant frequency for describing the chewing activity. The viscoelastic parameters loss modulus and loss factor decreased during aging only in the GIC, which is an indication of cement maturation, while they increased (or remained unchanged) in all resin-based materials, which is an indication of polymer plasticization. The evaluation of the viscoelastic behavior of these materials is of the utmost importance and should always be viewed as a supplement to already accepted analytical tests.

## Figures and Tables

**Table 1:** Analyzed luting materials: product name, batch number, and material type. All analyzed luting materials were manufactured by the company VOCO (Cuxhaven, Germany).

Luting Material	LOT	Material Type
Bifix QM	1905094	dual-cured RBC
Bifix SE	1910348	dual-cured, self-adhesive RBC
Meron	1906388	glass ionomer cement
Meron Plus QM	1908135	Resin-modified glass ionomer cement
Provicol QM Plus	1907633	temporary phosphate cement

**Table 2:** Three-point bending test: Weibull modulus *m* with standard error (Std. Error) and R square (*R*^2^) values, flexural strength *FS*, flexural modulus *FM* (mean *M* and standard deviation *SD*). Superscript (subscripts) letters indicate homogeneous groups in 24 h (TC) groups; Tukey’s post hoc test (α = 0.05).

Material	Aging	m	Std. Error	R^2^	FS		FM	
					M	SD	M	SD
Bifix QM	24 h	10.89	0.47	0.97	127.5 ^B^	14.2	5.4 ^A^	0.8
	TC	4.70	0.23	0.96	112.6 _a_	26.6	6.1 _a_	0.9
Bifix SE	24 h	15.61	0.82	0.95	138.2 ^A^	10.6	4.2 ^B^	0.5
	TC	2.31	0.24	0.83	81.1 _b_	25.3	3.1 _b_	0.5
Meron	24 h	3.10	0.12	0.97	15.3 ^D^	5.3	2.8 ^D^	0.7
	TC	2.69	0.13	0.96	7.1 _d_	2.8	2.9 _b_	0.5
Meron Plus QM	24 h	5.11	0.28	0.95	37.5 ^C^	8.8	1.3 ^C^	0.2
	TC	8.54	0.34	0.97	43.8 _c_	6.0	1.8 _c_	0.2

**Table 3:** Fracture toughness arranged in descending order. Superscript characters indicate statistically homogeneous subgroups within a column; Tukey’s honestly significant difference (HSD) test, α = 0.05.

Luting Material	Fracture Toughness, MPa·m^1/2^	
	Mean	Standard Deviation
BifixSE	1.19 ^a^	0.13
Bifix QM	0.72 ^b^	0.13
MeronQM plus	0.24 ^c^	0.06
Meron	0.17 ^c^	0.04
Provicol	0.00 ^d^	0.00

## Data Availability

Data is available on request.
